# Survival Advantage Associated with Decrease in Stage at Detection from Stage IIIC to Stage IIIA Epithelial Ovarian Cancer

**DOI:** 10.1155/2014/312193

**Published:** 2014-09-01

**Authors:** John Hoff, Lauren Baldwin, Jason Lefringhouse, Edward Pavlik, Rachel Miller, Christopher DeSimone, Frederick Ueland, Thomas Tucker, Richard Kryscio, J. R. van Nagell

**Affiliations:** ^1^Division of Gynecologic Oncology, Department of Obstetrics & Gynecology, Markey Cancer Center, University of Kentucky, 800 Rose Street, Lexington, KY 40536-0293, USA; ^2^Cancer Prevention and Control Program, Markey Cancer Center, University of Kentucky, Lexington, KY 40536-0293, USA; ^3^Department of Statistics, Markey Cancer Center, University of Kentucky, Lexington, KY 40536-0293, USA

## Abstract

*Objective.* The aim of this study was to document the survival advantage of lowering stage at detection from Stage IIIC to Stage IIIA epithelial ovarian cancer. *Methods.* Treatment outcomes and survival were evaluated in patients with Stage IIIA and Stage IIIC epithelial ovarian cancer treated from 2000 to 2009 at the University of Kentucky Markey Cancer Center (UKMCC) and SEER institutions. *Results.* Cytoreduction to no visible disease (*P* < 0.0001) and complete response to platinum-based chemotherapy (*P* < 0.025) occurred more frequently in Stage IIIA than in Stage IIIC cases. Time to progression was shorter in patients with Stage IIIC ovarian cancer (17 ± 1 months) than in those with Stage II1A disease (36 ± 8 months). Five-year overall survival (OS) improved from 41% in Stage IIIC patients to 60% in Stage IIIA patients treated at UKMCC and from 37% to 56% in patients treated at SEER institutions for a survival advantage of 19% in both data sets. 53% of Stage IIIA and 14% of Stage IIIC patients had NED at last followup. *Conclusions.* Decreasing stage at detection from Stage IIIC to stage IIIA epithelial ovarian cancer is associated with a 5-year survival advantage of nearly 20% in patients treated by surgical tumor cytoreduction and platinum-based chemotherapy.

## 1. Introduction

Despite advances in radical surgery, postoperative care, and chemotherapy, ovarian cancer remains the leading cause of gynecologic cancer mortality among women in the United States. This year, over 14,000 deaths from ovarian cancer will be reported in the United States alone [1]. Most women continue to present with advanced disease where the cost of treatment is high and survival is low. Since the 5-year survival of patients with early stage ovarian cancer is excellent, many investigators believe that the most effective way to reduce ovarian cancer mortality is through earlier detection. It has been estimated that if 75% of ovarian cancer cases were detected with early stage disease, the number of women dying of this cancer could be reduced by one half. Recent data from 3 of the 4 major ovarian cancer screening trials indicates that regular screening of high risk populations with a combination of serum [2–5] biomarkers and ultrasound is associated with a decrease in stage at detection. Specifically, a statistically higher percent of ovarian cancer patients detected through screening had Stage I or II disease when compared to ovarian cancer patients in control populations detected clinically [2–4] in the United Kingdom Collaborative Trial of Ovarian Cancer Screening (UKCTOCS), the Multicenter Japanese Trial, and the University of Kentucky Ovarian Cancer Screening (UKOCS) trial. In addition, a substage shift from Stage IIIC to Stage IIIA in cases detected by screening was reported in one of these trials [4]. The following investigation was undertaken to document the survival advantage associated with reducing substage at detection from Stage IIIC to Stage IIIA epithelial ovarian cancer in the era of primary tumor cytoreduction followed by platinum-based chemotherapy.

## 2. Methods

This investigation was undertaken after approval of the University of Kentucky Human Subjects Institutional Review Board. All patients with FIGO Stage IIIA and Stage IIIC epithelial ovarian cancer treated from 2000 to 2009 were identified from the UKMCC Tumor Registry and the SEER 18 Cancer Registry. Data abstracted from both registries included stage and substage at detection, cell type of epithelial ovarian cancer, age at diagnosis, number of live births at diagnosis, race, and geographic location (Appalachian versus non-Appalachian), and being urban versus being rural. In addition, hospital and outpatient records were reviewed on all patients treated at the UKMCC in order to determine the extent of surgical tumor cytoreduction, response to chemotherapy, time to disease progression, and sites of recurrence. Patients treated at the UKMCC all underwent standard surgical staging including total abdominal hysterectomy, bilateral salpingo-oophorectomy, omentectomy, and pelvic/paraaortic lymph node sampling. Bowel resection, splenectomy, and other upper abdominal surgeries were performed on a case by case basis, in an attempt to achieve maximal tumor cytoreduction. Complete debulking was defined as no visible residual tumor after surgical cytoreduction. Tumors were classified histologically according to the World Health Organization system and were staged according to the International Federation of Gynecology and Obstetrics (FIGO) Staging System (Table 1) [6]. Following surgery, patients were entered on Institutional or Gynecologic Oncology Group (GOG) Treatment Protocols, which usually included a minimum of 6 cycles of platinum-based chemotherapy. Patients were examined clinically at monthly intervals during chemotherapy, every 3 months for the next 2 years, and every 6 months thereafter. Ca 125 biomarker determinations were obtained at the time of clinical examination, and CT scans of the chest, abdomen, and pelvis were performed when indicated at 6-month intervals. Selected patients with an abnormality on CT scan or doubling serum biomarker levels underwent PET scans and CT-directed needle biopsies to confirm the presence of recurrent disease.

Criteria for tumor response and progression were those recommended by the Response Evaluation Criteria in Solid Tumors (RECIST) Committee [7, 8]. Complete response (CR) was defined as normal clinical biomarker levels and no clinical or radiographic evidence of disease, and partial response (PR) was defined as at least a 30% decrease in the sum of the longest measurable tumor diameter as measured by CT scan, MRI, or X-ray. Tumor progression was defined as at least a 20% increase in the sum of the longest measurable tumor diameter, or greater than a doubling of serum Ca-125 in the presence of radiographically identifiable disease [8]. Time to progression was defined as the time from primary diagnosis to documented disease progression. Platinum sensitivity was determined 6 months from completion of chemotherapy. Patients classified as platinum-sensitive had a CR or PR to primary platinum therapy and no evidence of progression within 6 months of completing chemotherapy. Platinum-resistant patients had less than a PR to primary platinum therapy or had a CR or PR and experienced tumor progression less than 6 months after completing chemotherapy. Platinum-sensitive patients experiencing tumor recurrence were treated again with platinum-based chemotherapy, whereas platinum-resistant patients were treated with single agent chemotherapy on an individual basis. Patient follow-up was coordinated by the UKMCC Institutional Cancer Registry, the American Cancer Society, and the Kentucky State Department of Vital Statistics. Overall survival (OS) was defined as the time from primary diagnosis to death from any cause. Survivors were censored on the last date they were known to be alive.

## 3. Statistical Analysis

All analyses were conducted using SAS software version 9.2 (SAS Institute Inc., Cary, NC, USA, 2008); tests were two-sided and *P* values ≤0.05 were considered statistically significant. The chi-square test was used to compare categorical data and the independent samples *t*-test for comparing continuous data between subgroups. Time-to-event data were analyzed using the Kaplan-Meier method and the log-rank test was used to compare survival distributions between groups.

## 4. Results

From 2000 to 2009, 15 patients with Stage IIIA and 186 patients with Stage IIIC epithelial ovarian cancer were treated at the UKMCC. Five of the patients with Stage IIIA ovarian cancer and 7 of the patients with Stage IIIC ovarian cancer were detected by screening. Demographic data for patients treated at the UKMCC from 2000 to 2009 are presented in Table 2. There were no significant differences in age at detection, race, geographic location, or ovarian cancer cell type in patients with Stage IIIA disease versus those with Stage IIIC disease. During this time period, 794 patients with Stage IIIA and 11,967 patients with Stage IIIC ovarian cancer were treated at 18 institutions in the SEER database (Table 3). There were no significant demographic differences in between patients with Stage IIIA and Stage IIIC ovarian cancer treated at SEER institutions. However, there was a slightly higher frequency of endometrioid and mucinous cell types in patients with Stage IIIA disease and a slightly higher frequency of serous cell types in those with Stage IIIC disease.

Outcomes of patients with Stages IIIA and IIIC ovarian cancer treated at the UKMCC from 2000 to 2009 are presented in Table 4. Surgical cytoreduction to no visible disease was achieved in 100% of Stage IIIA patients and in 34.4% of Stage IIIC patients (*P* < 0.0001). Sixty-five percent of patients with Stage IIIC ovarian cancer had <1 cm residual disease after tumor debulking. All patients with Stage IIIA ovarian cancer experienced a complete response (CR) to platinum-based chemotherapy (as defined by no radiologic or biomarker evidence of disease after 6 cycles of chemotherapy) versus 74.2% of patients with Stage IIIC disease (*P* < 0.025). As expected, the most common sites of recurrence in both stage groups were intraperitoneal, but 12% of Stage IIIC cases also developed extraperitoneal spread. The most common sites of extraperitoneal recurrence were lung (10), liver (6), brain (2), and inguinal lymph nodes (2). The mean time to progression was significantly shorter (*P* < 0.01) in patients with Stage IIIC disease (17 ± 1.3 months) than in patients with Stage IIIA disease (36 ± 8 months).

**Figure 1:**
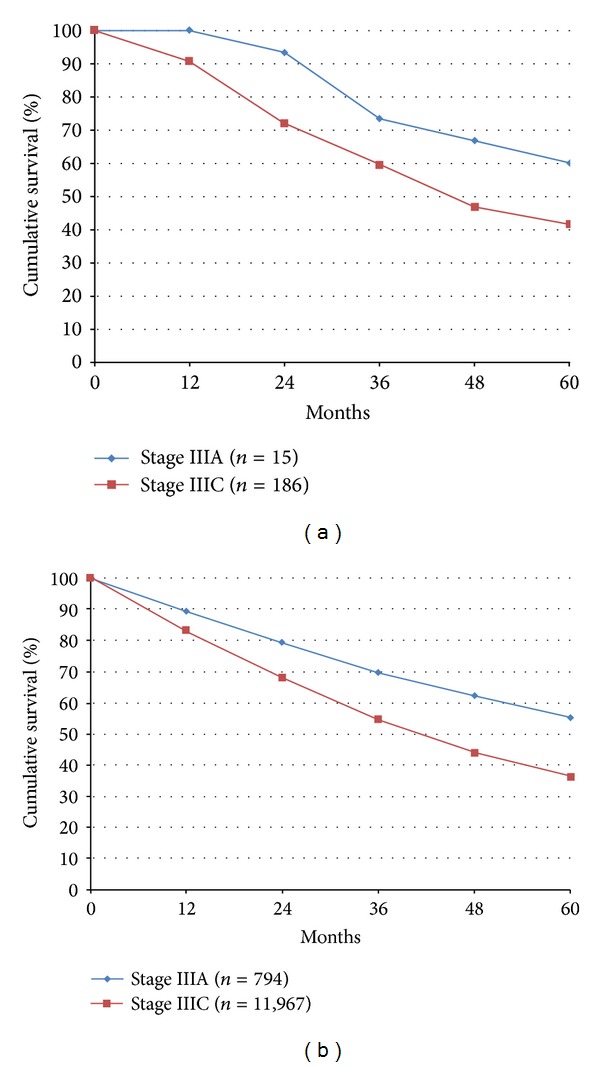
(a) Overall survival of Stage IIIA versus Stage IIIC epithelial ovarian cancer patients at the University of Kentucky Markey Cancer Center (2000–2009). (b) Overall survival of Stage IIIA versus Stage IIIC epithelial ovarian cancer patients in SEER∗18 Registries (2000–2009).

The overall survival (OS) of patients with Stages IIIA and IIIC epithelial ovarian cancer treated at the UKMCC and the SEER institutions is presented in Figure 1. The 5-year OS of patients with Stage IIIA disease treated at the UKMCC was 60% compared to 41% in patients with Stage IIIC disease (Figure 1(a)). Moreover, 53% of patients with Stage IIIA ovarian cancer had no evidence of disease at the time of their last follow-up visit versus 14.5% of patients with Stage IIIC ovarian cancer (*P* < 0.0008). The 5-year OS of Stage IIIA ovarian cancer patients treated at SEER institutions was 56% compared to 37% in Stage IIIC cases (Figure 1(b)). Therefore, the 5-year survival advantage associated with downstaging IIIC to IIIA ovarian cancer was 19% both in patients treated at the UKMCC and in those treated at the SEER institutions. The small number of patients with Stage IIIA ovarian cancer treated at the UKMCC (*N* = 15) limited meaningful survival analysis. However, the same 19% survival advantage observed between Stage IIIA cases and Stage IIIC cases treated at SEER institutions was highly significant (*P* < 0.0001) when a greater number of patients were included in both Stage III subgroups.

The 5-year OS of UKMCC patients with completely debulked Stage IIIC ovarian cancer was 64.8%. This is similar to the OS of UKMCC patients with Stage IIIA ovarian cancer and significantly higher (*P* < 0.0001) than the 29.7% survival of UKMCC patients with incompletely debulked Stage IIIC disease.

## 5. Discussion

Sonographic and biomarker screening of asymptomatic women at high risk for ovarian cancer has been initiated in several countries as a means to lower stage at diagnosis [2–5]. Also, tumor morphology indexing and serum biomarker profiling have been used to identify ovarian tumors at the highest risk of malignancy so that women with these tumors can be referred to tertiary care centers for their surgery [9–13]. The goal of these efforts is to promote earlier detection of ovarian cancer and to enable patients with these malignancies to be treated earlier in the disease process by gynecologic oncologists. The rationale of these approaches is based on the reported excellent 5-year survival rate of patients with early stage ovarian cancers and the improved outcomes of those receiving appropriate surgery and chemotherapy [14]. Although the effect of screening on ovarian cancer mortality has not yet been answered definitively, the efficacy of screening tests to detect early stage disease in asymptomatic women is well documented. For example, the UKCTOCS [2] screened 202,638 postmenopausal women and reported that 47% of ovarian cancer patients detected by screening had Stage I or II disease versus 26% in the unscreened control population (*P* < 0.005) [2]. Similarly, 63% of ovarian cancer patients detected by screening in the Multicenter Japanese Trial had Stage I disease versus 38% in the control group [3].

In a recent report from the ongoing UKOCS trial, not only were 68% of ovarian cancer patients diagnosed with Stage I or II disease, but there was also a decrease in substage at detection in patients with Stage III disease [4]. Specifically, of the 14 screen-detected patients with Stage III ovarian cancer, 5 patients (36%) had Stage IIIA disease, and 3 patients (21%) had Stage IIIB disease. As a result, the 5-year survival of Stage III cases detected by screening was significantly higher than that of clinically detected Stage III cases, 84% of whom had Stage IIIC disease.

Findings of the present investigation confirm a significant outcomes benefit to patients when stage at detection is reduced from Stage IIIC to IIIA. In patients with Stage IIIA disease, there was a statistically significant increase in the frequency of successful tumor debulking, complete response to platinum-based chemotherapy, and disease-free status. As a result, there was a significant survival advantage of almost 20% in patients with Stage IIIA ovarian cancer when compared to those with Stage IIIC disease in both the SEER and UKMCC groups. Since one-third of the patients with Stage IIIA ovarian cancer in the UKMCC series were detected by screening, lead time bias may have contributed to the observed increase in survival noted. However, this bias would not be apparent in the SEER cases since all patients were detected clinically. Also, there may be a biologic difference in ovarian cancer according to substage in patients treated at SEER institutions since there was a higher frequency of endometrioid cancer in Stage IIIA cases and a higher frequency of serous cancers in those with Stage IIIC disease.

The prognostic effect of complete tumor debulking was noted in this study and confirmed the findings of several prior investigations [15–18]. The 5-year OS of completely debulked UKMCC Stage IIIC cases was 64.8% as compared to 29.7% in patients with visible residual disease after cytoreductive surgery. Interestingly, the 5-year OS of completely debulked Stage IIIC cases was approximately the same as the 60% OS of Stage IIIA cases observed in this study. This is consistent with the observations of Le et al. [19], who reported that 81 completely cytoreduced patients with Stage IIIB-IIIC ovarian cancer had essentially the same survival as 24 Stage IIA-IIIA completely cytoreduced patients with no visible extrapelvic disease. Similarly, Eisenkop et al. [17], in a study of 408 patients with Stage IIIC ovarian cancer, concluded that the completeness of surgical cytoreduction had a more significant effect on prognosis than the extent of metastatic disease prior to surgery. Our observations, however, are somewhat at variance with those of Hoskins et al. [20]. These authors stratified 349 patients with Stage III ovarian cancer, all of whom were cytoreduced to ≤1 cm residual disease, according to extent of disease prior to surgery. Patients with >1 cm extrapelvic disease before surgery had a median survival of 31 months, whereas those with ≤1 cm extrapelvic disease before surgery had a median survival of 51 months. These authors concluded that the innate biological properties of ovarian cancer play a more important role in determining prognosis than the extent of surgical cytoreduction.

Although the present investigation is retrospective, it does provide a detailed comparison of patients with Stages IIIA and IIIC epithelial ovarian cancer, all of whom received complete surgical staging, tumor debulking, and platinum-based chemotherapy during the same time period by gynecologic oncologists at one institution. The number of patients with clinically detected Stage IIIA ovarian cancer remains small, thereby prolonging the time requirement necessary to complete a prospective comparison of patients within Stage III. In the present study, however, analysis of data from SEER institutions allowed outcomes of a large number of patients with Stage IIIA ovarian cancer to be compared to those of an even greater number of patients with Stage IIIC disease. Importantly, the improvement in survival from Stage IIIC to Stage IIIA was identical in both data sets and was highly significant in the SEER experience.

The findings of this investigation confirm a significant survival benefit of earlier detection in epithelial ovarian cancer, not only by increasing the frequency of patients with early stage disease, but also by diagnosing Stage III cancers at an earlier substage. The 19% 5-year survival advantage observed in patients with Stage IIIA versus Stage IIIC ovarian cancer is significant and should encourage further research into methods to improve the earlier detection of this disease.

## Figures and Tables

**Table 1:** Definitions of Stage IIIA and Stage IIIC ovarian cancer∗.

Stage III	
Tumor involves one or both ovaries with cytologically or histologically confirmed spread to the peritoneum outside the pelvis and/or metastasis to the retroperitoneal lymph nodes.	
Stage IIIA	
Microscopic metastasis beyond the pelvis.	
Stage IIIC	
Macroscopic, extrapelvic, peritoneal metastasis >2 cm in the greatest dimension and/or regional lymph node metastasis.	

*From Edge et al. [6].

**Table 2:** Demographic data Stage IIIA versus Stage IIIC epithelial ovarian cancer, University of Kentucky Markey Cancer Center 2000–2009.

	IIIA	IIIC	Significance
	Mean	Mean	

Age at diagnosis	58.6 ± 15.9	58.2 ± 12.4	*P* = 0.89
Number of live births at diagnosis	1.9 ± 2.1	2.1 ± 1.6	*P* = 0.54

	*N*	*N*	

Race			
Black	0	1 (0.5%)	
White	15 (100%)	185 (99.5%)	*P* = 0.78
Other	0	0	
Appalachian Region			
Appalachia	8 (53%)	115 (62%)	*P* = 0.52
Non-Appalachia	7 (47%)	71 (38%)	
Urban region			
Rural	5 (33%)	70 (38%)	*P* = 0.74
Urban	10 (67%)	116 (62%)	
Cell type			
Serous carcinoma	8 (54%)	118 (63%)	
Endometrioid carcinoma	3 (20%)	17 (10%)	*P* = 0.46
Mixed adenocarcinoma (NOS)	2 (13%)	36 (19%)	
Clear cell, mucinous, carcinoma (NOS)	2 (13%)	15 (8%)	

NOS: not otherwise specified.

**Table 3:** Demographic data Stage IIIA versus Stage IIIC epithelial ovarian cancer, US SEER 18 Registries 2000–2009.

Stage	IIIA	IIIC	Significance
Number	794	11,967	
Age at diagnosis (mean)	63.2 ± 14.4	62.2 ± 14.8	*P* = 0.42

	*N*	*N*	

Race			
Black	49 (6.2%)	712 (5.9%)	*P* = 0.18
White	680 (85.6%)	10,456 (87.4%)	
Other	65 (8.2%)	799 (6.7%)	
Appalachian Region			
Appalachia	30 (3.8%)	539 (4.5%)	*P* = 0.34
Non-Appalachia	764 (96.2%)	11,428 (95.5%)	
Urban region			
Rural	78 (9.8%)	1,199 (10.0%)	*P* = 0.86
Urban	716 (90.2%)	10,768 (90.0%)	
Cell type			
Serous carcinoma	396 (50.0%)	7,952 (66.4%)	*P* = 0.05
Endometrioid carcinoma	90 (11.3%)	740 (6.2%)	
Mixed adenocarcinoma (NOS)	32 (4.0%)	437 (3.7%)	
Mucinous carcinoma	91 (11.5%)	1,282 (10.7%)	
Carcinoma (NOS), other	185 (23.2%)	1,556 (13.0%)	

NOS: not otherwise specified.

**Table 4:** Treatment outcomes Stage IIIA versus Stage IIIC epithelial ovarian cancer, University of Kentucky Markey Cancer Center 2000–2009.

	IIIA	IIIC	Significance
	(*N* = 15)	(*N* = 186)	
Complete debulking (no visible residual disease)	15 (100%)	64 (34.4%)	*P* < 0.0001
Complete response to chemotherapy	15 (100%)	138 (74.2%)	*P* = 0.025
Time to progression			
Median (months)	29	13	
Mean (months)∗	36 ± 8	17 ± 1.3	*P* < 0.01
Range (months)	15–75	2–123	
Site of recurrence			
Intraperitoneal	7 (46.7%)	137 (73.7%)	
Intra- + extraperitoneal	0	8 (4.3%)	
Extraperitoneal	0	14 (7.5%)	
NED∗∗	8 (53.3%)	27 (14.5%)	*P* < 0.008
Overall survival			
2 years	93.3%	72.0%	*P* < 0.07
5 years	60.0%	41.8%	

*Mean ± standard error of mean.

**NED: no evidence of disease.
